# A Genetic Screen for Dominant Enhancers of the Cell-Cycle Regulator α-Endosulfine Identifies Matrimony as a Strong Functional Interactor in *Drosophila*

**DOI:** 10.1534/g3.111.001438

**Published:** 2011-12-01

**Authors:** Jessica R. Von Stetina, Kimberly S. LaFever, Mayer Rubin, Daniela Drummond-Barbosa

**Affiliations:** * Department of Cell and Developmental Biology, Vanderbilt University Medical Center, Nashville, TN 37232; † Department of Biochemistry and Molecular Biology; ‡ Environmental Health Sciences, Division of Reproductive Biology, Johns Hopkins Bloomberg School of Public Health, Baltimore, MD 21205

**Keywords:** α-endosulfine, matrimony, polo, early embryonic cell cycle, *Drosophila*

## Abstract

The coordination of cell-cycle events with developmental processes is essential for the reproductive success of organisms. In *Drosophila melanogaster*, meiosis is tightly coupled to oocyte development, and early embryos undergo specialized S-M mitoses that are supported by maternal products. We previously showed that the small phosphoprotein α-endosulfine (Endos) is required for normal oocyte meiotic maturation and early embryonic mitoses in *Drosophila*. In this study, we performed a genetic screen for dominant enhancers of *endos^00003^* and identified several genomic regions that, when deleted, lead to impaired fertility of *endos^00003^/+* heterozygous females. We uncovered *matrimony* (*mtrm*), which encodes a Polo kinase inhibitor, as a strong dominant enhancer of *endos*. *mtrm^126^ +/+ endos^00003^* females are sterile because of defects in early embryonic mitoses, and this phenotype is reverted by removal of one copy of *polo*. These results provide compelling genetic evidence that excessive Polo activity underlies the strong functional interaction between *endos^00003^* and *mtrm^126^*. Moreover, we show that *endos* is required for the increased expression of Mtrm in mature oocytes, which is presumably loaded into early embryos. These data are consistent with the model that maternal *endos* antagonizes Polo function in the early embryo to ensure normal mitoses through its effects on Mtrm expression during late oogenesis. Finally, we also identified genomic deletions that lead to loss of viability of *endos^00003^/+* heterozygotes, consistent with recently published studies showing that *endos* is required zygotically to regulate the cell cycle during development.

Precise control of the cell-cycle machinery at specific developmental stages is crucial to ensure appropriate cellular outcomes, and research in *Drosophila melanogaster* has led to significant advances in understanding how this regulation is achieved (Lee and Orr-Weaver 2003). Two relevant examples of coordination between the cell cycle and development are found in oocyte meiotic maturation during oogenesis and in the specialized early embryonic mitoses.

*Drosophila* oocytes develop within egg chambers, or follicles, which progress through 14 stages of development within ovarian subunits termed ovarioles (Spradling 1993). Each egg chamber is composed of an inner germline cyst containing one oocyte and 15 supportive nurse cells and is surrounded by a monolayer of somatic follicle cells. The oocyte initiates meiosis within very early cysts before the acquisition of follicle cells but remains arrested in prophase of meiosis I through most of oogenesis (Figure 1A, A′; King 1970). During the oocyte arrest in prophase I, the egg chamber goes through most of its development, including the dumping of nurse cell cytoplasmic contents into the growing oocyte during stage 11 (Spradling 1993). At stage 13, as the remaining nurse cell nuclei are gradually eliminated by cell death, the fully grown oocyte undergoes meiotic maturation, a process whereby the prophase I arrest is released and the oocyte progresses through a second arrest in metaphase I (Figure 1B, B′, B′′). The metaphase I arrest is maintained in mature stage 14 oocytes until egg activation takes place as the oocyte passes through the oviduct during egg laying (Horner and Wolfner 2008).

**Figure 1:**
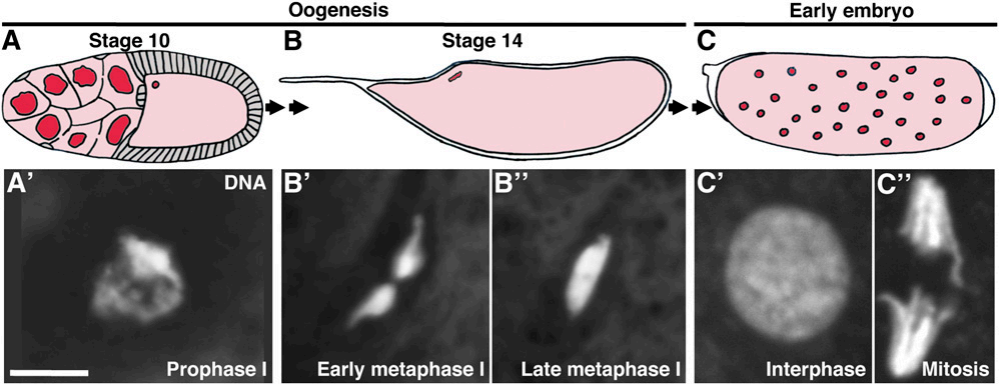
Coordination between the cell cycle and development during meiotic maturation and early embryonic mitoses. (A) Stage 10 egg chamber diagram, exemplifying a stage in which the oocyte is arrested in prophase I. (B) Diagram of a stage 14 oocyte, which has progressed to metaphase I as a result of meiotic maturation. (C) Early embryo showing nuclei that undergo mitotic divisions in a shared cytoplasm, relying on maternal stockpiles of RNA and proteins. Stage 10 follicle cells are shown in gray, germline and early embryo cytoplasm in pink, and nuclei in red. Double arrows between (A) and (B) represent egg chamber development and oocyte meiotic maturation at stage 13. Double arrows between (B) and (C) represent completion of meiosis (upon egg activation) and fertilization. DAPI-stained DNA from a stage 10 oocyte arrested in prophase I (A′), a stage 14 oocyte in early (B′) or late (B′′) metaphase I, and from early embryonic nuclei in interphase (C′) or mitosis (C′′) are shown below corresponding diagrams. Scale bar, 5 μm.

Embryonic development, which ensues after egg activation and fertilization, relies initially on maternal RNA and proteins loaded into the oocyte during oogenesis, independently of zygotic transcription (Lee and Orr-Weaver 2003). Specifically, the first 13 embryonic cell cycles, which occur within a common, syncytial cytoplasm, are maternally controlled and represent variant mitotic cell cycles that lack gap (G) phases and simply alternate between DNA synthesis (S) and mitosis (M; Figure 1C, C′, C′′). Subsequently, interphase lengthens by the addition of G2 and then G1 phases, and zygotic transcription becomes essential (Lee and Orr-Weaver 2003).

Our previous studies uncovered critical roles for the small phosphoprotein α-endosulfine (Endos) in both oocyte meiotic maturation and early embryonic mitoses in *Drosophila* (Von Stetina *et al.* 2008). Oocytes in *endos^00003^* homozygous mutant females have prolonged prophase I arrest and abnormal nuclear envelope breakdown, and they fail to progress into metaphase I. In addition, the rare resulting embryos that initiate development display abnormal DNA and spindle morphologies during early, maternally controlled mitoses (Von Stetina *et al.* 2008). Consistent with these cell-cycle defects, *endos^00003^* homozygous mutant oocytes have markedly low levels of *in vivo* MPM2 phosphoepitopes, which result from phosphorylation of targets of the Polo and Cdk1 cell-cycle regulatory kinases. Paradoxically, however, *endos^00003^* homozygous mutant oocytes show normal levels of Cdk1 kinase activity in *in vitro* assays (Von Stetina *et al.* 2008). Nevertheless, recent biochemical studies using *Xenopus* egg extracts showed that upon its phosphorylation by Greatwall (Gwl) kinase, the vertebrate homolog of Endos binds to and inhibits protein phosphatase 2A, a Cdk1 antagonist that dephosphorylates its targets (Gharbi-Ayachi *et al.* 2010; Mochida *et al.* 2010). In addition, *endos* was very recently shown to control *Drosophila* cell cycles through a similar pathway (Rangone *et al.* 2011), potentially explaining our paradoxical findings.

Despite these recent advances, Endos likely has additional molecular functions that contribute to cell-cycle regulation. For example, in an *in vitro* screen for Endos binding partners, two specific interactors were identified, including the E3 ubiquitin ligase Elgi. Interestingly, although mutation of *elgi* results in premature meiotic maturation, Elgi does not appear to target Endos for ubiquination or to mediate the effects of Endos on MPM2 epitope levels (Von Stetina *et al.* 2008). A complete understanding of the diverse molecular roles of Endos will require the identification of additional genes that function with this small regulatory protein in the control of meiotic maturation and/or early embryonic mitoses.

Here, we describe a genetic screen for dominant enhancers of *endos^00003^* heterozygotes. We reasoned that halving the gene dosage of *endos* may sensitize females to reduced dosage of other genes functioning with *endos* to control meiotic maturation and/or early embryonic mitoses, leading to reduced fertility of double heterozygous females. We screened approximately 200 available deficiencies and identified 11 genomic regions that when deleted dominantly lead to sterility or semi-sterility of *endos^00003^* heterozygous females. Our screen also identified five genomic regions that result in lethality of *endos^00003^* heterozygotes, uncovering a zygotic function of *endos*. Further analyses of an enhancer reducing the fertility of *endos^00003^* females revealed that *matrimony* (*mtrm*), a negative regulator of Polo kinase (Xiang *et al.* 2007), functions together with Endos to regulate early embryonic mitoses. Specifically, we find that *mtrm^126^* +/+ *endos^00003^* double-heterozygous females have markedly reduced fertility relative to single heterozygotes because of defects in early, maternally controlled embryonic mitoses, and that this strong genetic interaction is likely due to the fact that Endos regulates Mtrm levels during late oogenesis.

## Materials and Methods

### *Drosophila* culture

*Drosophila* stocks and crosses were maintained at 22-25° on standard medium. *y w* was used as a wild-type control. *endos^00003^* is a strong hypomorphic allele caused by a *P* element insertion in its 5′ untranslated region (Drummond-Barbosa and Spradling 2004; Von Stetina *et al.* 2008). *mtrm^126^* is a molecular null allele (Xiang *et al.* 2007); in all crosses, *mtrm^126^* was introduced into the analyzed genotypes via the male because this stock is contaminated with *Wolbachia*, a maternally transmitted intracellular bacterium (Serbus *et al.* 2008). *polo^16-1^* is a strong hypomorph (Donaldson *et al.* 2001; Roseman *et al.* 1995), and the null *elgi^1^* allele has been described (Von Stetina *et al.* 2008). Deficiency Kit stocks were obtained from the Bloomington *Drosophila* Stock Center (http://flystocks.bio.indiana.edu/). The *GFP::polo* transgene carries a fully functional *polo* gene within a 7-kb genomic fragment with the *GFP* coding sequence fused immediately upstream of and in frame with the *polo* coding region (Moutinho-Santos *et al.* 1999). Balancer chromosomes and other genetic elements are described in FlyBase (http://flybase.org).

For measurement of embryo hatch rates, females of various genotypes were crossed to *y w* males, and embryos collected overnight. For most genotypes, 100 to 150 collected embryos were placed in groups of 10 on molasses plates containing a small amount of wet yeast in their center. (For genotypes displaying low rates of egg laying, fewer embryos were collected.) Plates were incubated in a humid chamber at 25° for 2 d, and unhatched embryos were counted and subtracted from the total to determine the hatch rate as a percentage. Experiments were performed in triplicate, and statistical analysis was performed using the Student’s *t* test.

### Deficiency screen

To identify deficiencies that lead to female sterility or lethality of *endos^00003^*/+ heterozygotes, or *endos^00003^* dominant enhancers, we performed an F1 screen (supporting information, Figure S1). *endos^00003^/TM3, Sb^1^* virgin females were crossed to males carrying 208 balanced deficiencies (*Df*; File S1) and progeny were analyzed. (We estimate that these deficiencies represent roughly 45% genome coverage because the entire Deficiency Kit from the Bloomington Stock Center contains 469 deficiencies, which together provide 98% coverage of the genome.) If no adult *endos^00003^/Df* or *Df/+; endos^00003^*/+ flies resulted from the cross (*i.e.*, only flies carrying balancer chromosomes were present among progeny), the genetic interaction was considered lethal. Otherwise, four *endos^00003^/Df* or *Df/+; endos^00003^/+* females were crossed to three *y w* males to test their fertility. If these females yielded drastically reduced or no progeny, the genetic interaction was considered semisterile or sterile, respectively. Only deficiencies that yielded reproducible results as *endos^00003^/+* enhancers in triplicate experiments were added to the final list of enhancer deficiencies. For each interacting genomic region identified through the deficiency screen, additional deficiencies were tested as shown in Table S1 and Table S3.

### Western blotting

For Western blotting analyses, ovaries or egg chambers were homogenized, electrophoresed, and transferred to membranes as described (Von Stetina *et al.* 2008). Membranes were blocked and probed with 1:500 guinea pig polyclonal anti-Mtrm (Xiang *et al.* 2007), 1:4,000 rat monoclonal anti-α-tubulin (YL1/2, Accurate Chemical & Scientific Corporation), or 1:50 mouse monoclonal anti-Actin (JLA20, Developmental Studies Hybridoma Bank). IRDye 800-conjugated goat anti-guinea pig (Rockland) or horseradish peroxidase-conjugated goat anti-rabbit, donkey anti-guinea pig, or goat anti-rat (Jackson ImmunoResearch Laboratories) secondary antibodies were used at 1:5000 or 1:4000 dilutions. The Odyssey Infrared Imaging System (LI-COR Biosciences) or enhanced chemiluminescence (Amersham Life Science) was used for detection.

### Immunostaining and fluorescence microscopy

Egg chamber staging and analysis of oocyte meiotic maturation were performed as described (Von Stetina *et al.* 2008), with some modifications. In brief, ovaries were dissected, fixed, and stained in 0.5 μg/ml 4′,6-diamidino-2-phenylindole (DAPI; Sigma-Aldrich) for 20 min. Analysis of DNA and spindles in early embryonic mitoses were performed essentially as described (Von Stetina *et al.* 2008). In brief, 0- to 60-min embryos were collected, dechorionated, shaken vigorously for 2 min in 1:1 heptane:methanol, fixed in methanol overnight, and stained with anti-α-tubulin FITC-conjugated antibody (DM1A clone; Sigma-Aldrich) at 1:300 dilution and 0.5 μg/ml DAPI or 10 μg/ml propidium iodide following RNAse A treatment. Samples mounted in Vectashield (Vector Laboratories) were imaged with a Zeiss Axioplan 2 or AxioImager-A2 fluorescence microscope, or LSM700 confocal microscope. For statistical analysis, results were subjected to the Yates’ chi-square test (χ^2^= Σ(|*O-E*|− 1/2)^2^/ *E*) (Yates 1934).

## Results

### A deficiency screen for dominant enhancers of *endos* heterozygosity identifies multiple interacting genomic regions

**Figure 2:**
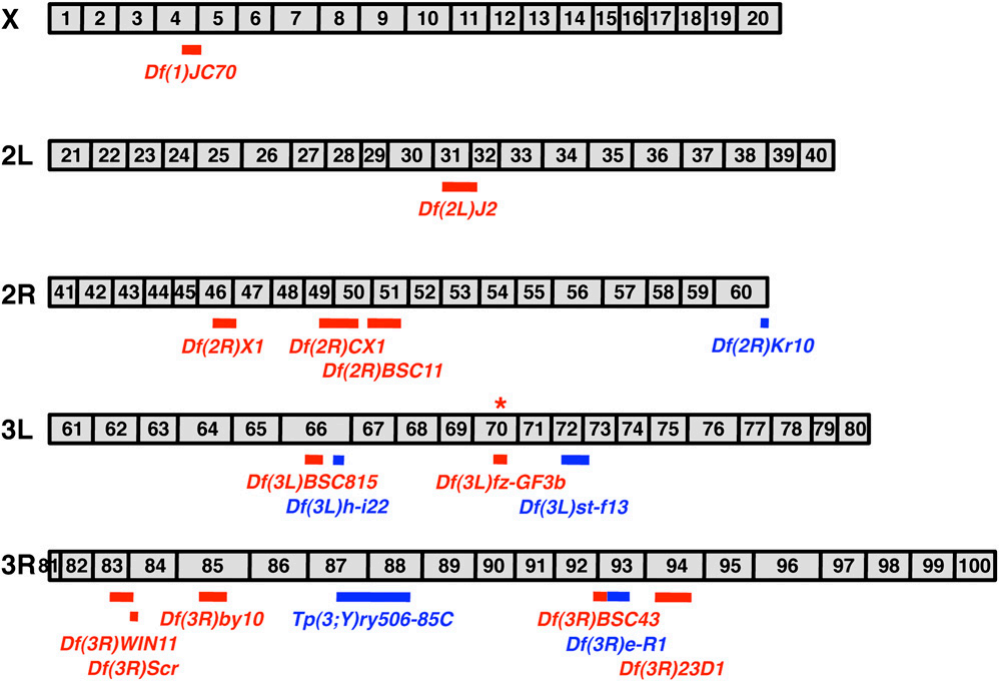
Chromosomal distribution of deficiencies that dominantly enhance *endos^00003^/+*. Gray bars depict chromosome arms (X, 2L, 2R, 3L, and 3R), and black numbers indicate polytene chromosome divisions. Deficiencies shown in red dominantly cause sterility or semisterility of *endos^00003^* heterozygous females, whereas those shown in blue dominantly cause lethality of *endos^00003^* heterozygous animals. Red and blue bars above deficiency indicate their deleted genomic regions according to FlyBase (http://flybase.org). Asterisk indicates *endos* location.

The small phosphoprotein Endos has critical biological roles in *Drosophila* oocyte meiotic maturation and early embryonic mitoses (Von Stetina *et al.* 2008). Although the molecular mechanisms of action of Endos during cell cycle regulation are likely complex, recent biochemical studies uncovering an *in vitro* role for the *Xenopus* homolog as a stoichiometric binding inhibitor of protein phosphatase 2A (Gharbi-Ayachi *et al.* 2010; Mochida *et al.* 2010) suggest that the gene dosage of *endos* might be relevant for its roles in the cell cycle. It is therefore conceivable that although *endos^00003^/+* heterozygous females are largely phenotypically normal (Drummond-Barbosa and Spradling 2004), removal of one copy of other genes that function together with *endos* in the regulation of meiotic maturation and/or early embryonic mitoses might genetically enhance *endos^00003^*, resulting in sterility or semisterility of those females. We performed an F1 screen for enhancers of *endos^00003^* heterozygous females using 208 deficiencies (∼45% genome coverage) from the Bloomington Stock Center Deficiency Kit (Figure S1 and File S1). As expected, *Df(3L)fz-GF3b*, which uncovers the *endos* gene itself, led to complete female sterility in combination with *endos^00003^*. We also found 11 additional deficiencies that impair the fertility of *endos^00003^* heterozygous females (Figure 2 and Table 1), and for all of the regions uncovered by these interacting deficiencies (except for *Df(3L)BSC815*; see below) additional deficiencies were tested to refine the results (Table S1).

**Table 1:** Deficiencies that reduce the fertility of *endos^00003^/*+ heterozygous females

Deficiency	Deleted segment*^a^*	Hatch rate*^b^*
*Df(1)JC70*	4C11—5A4	21 ± 11 (416)
*Df(2L)J2*	31B1—32A2	n.d.
*Df(2R)X1*	46C2—47A1	n.d.
*Df(2R)CX1*	49C1—50D5	41 ± 2.0 (300)
*Df(2R)BSC11*	50E6—51E4	3.3 ± 3.2 (300)
*Df(3L)BSC815**^c^*	66C3—66D4	26 ± 3.6 (317)
*Df(3L)fz-GF3b**^d^*	70C2—70D5	0 ± 0 (344)
*Df(3R)WIN11*	83E1—84A5	53 ± 9.9 (21)*^e^*
*Df(3R)Scr*	84A1—84B2	n.d.
*Df(3R)by10*	85D8—85E13	26 ± 27 (243)
*Df(3R)BSC43*	92F7—93B6	94 ± 8.5 (54)*^e^*
*Df(3R)23D1*	94A3—94D4	46 ± 5.7 (298)

a Deleted genomic region represented according to polytene chromosome divisions (http://flybase.org).

b Percentage of embryos from *endos^00003^/Df* or *Df/*+*; endos^00003^/*+ females that hatch. Results from three or four experiments shown as mean ± SD. The total number of embryos analyzed is shown in parentheses.

c *Df(3L)BSC815* uncovers *mtrm*.

d *Df(3L)fz-GF3b* uncovers *endos*.

e Females lay very few eggs, which contributes to their reduced fertility.

Our screen also revealed a zygotic role for *endos*. Specifically, five deficiencies led to zygotic lethality of *endos^00003^* heterozygotes during development (Figure 2 and Table S2), and additional deficiencies uncovering the identified interacting regions were also tested (Table S3). These results are consistent with a recent study that showed that *endos* function is zygotically required for proper neuroblast cell cycles during *Drosophila* development (Rangone *et al.* 2011).

### *endos* and *mtrm* are strong genetic interactors

**Figure 4:**
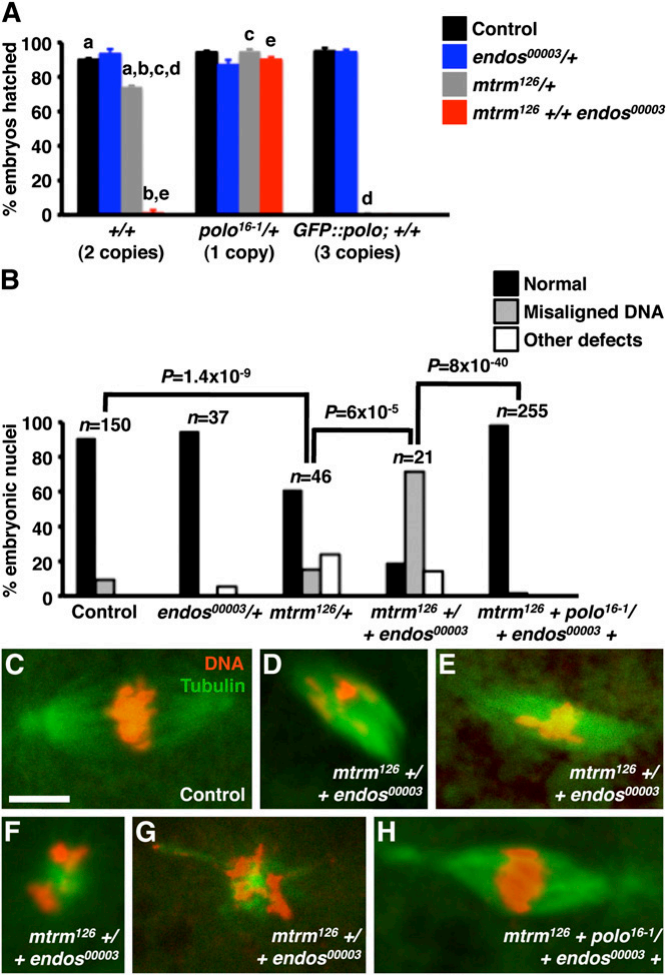
*endos* and *mtrm* show dominant genetic interactions during early embryonic mitoses. (A) Hatch rates for embryos derived from *y w* control, *endos^00003^/+*, *mtrm^126^/+*, or *mtrm^126^ +/+ endos^00003^* females in wild-type (2 copies of *polo*), *polo^16-1^/+* (one copy of *polo*) or *GFP::polo* (three copies of *polo*) background. Three hundred embryos were analyzed per genotype. a *vs.* a, *P* = 2.8 × 10^−7^. b *vs.* b, *P* = 6.8 × 10^−76^. c *vs.* c, *P* = 2.1 × 10^−12^. d *vs.* d, *P* = 2.5 × 10^−78^. e *vs.* e, *P* = 4.3 × 10^−106^. (B) Quantification of percentage of nuclei from embryos derived from *y w* control, *endos^00003^/+*, *mtrm^126^/+*, or *mtrm^126^ +/+ endos^00003^*, or *mtrm^126^ + polo^16-1^/+ endos^00003^ +* females displaying normal or defective DNA and spindle morphology. In addition to misaligned DNA, other defects include attached spindles, highly condensed DNA, or no mitotic nuclei with two rosettes present. Numbers of nuclei (n) analyzed are shown above bars. Ten to fifteen embryos were analyzed for each genotype. (C–H) Examples of *y w* control embryo nucleus with normal DNA morphology (C), or nuclei from embryos produced by *mtrm^126^ +/+ endos^00003^* female showing misaligned DNA and abnormal spindle (D, E) or highly condensed and disorganized DNA (F,G), and normal nucleus from embryo produced by *mtrm^126^ + polo^16-1^/+ endos^00003^ +* female (H) are shown. Scale bar, 5 μm.

*Df(3L)BSC815*, which removes region 66C3−66D4, drastically reduced the fertility of *endos^00003^*/+ heterozygous females, with hatch rates of 26% for embryos produced by these females (Table 1). Region 66C3−66D4 contains *matrimony* (*mtrm*), a gene previously reported to encode a negative regulator of Polo kinase that controls the timing of meiotic maturation and chromosome segregation in a dose-sensitive manner. Females that are heterozygous for the null *mtrm^126^* allele display premature nuclear envelope breakdown (a hallmark of meiotic maturation) and increased frequencies of X and 4th chromosome nondisjunction, and these phenotypes are completely suppressed by removal of one copy of *polo* (Xiang *et al.* 2007). To test whether the interaction between *endos^00003^* and *Df(3L)BSC815* was caused by the loss of one copy of *mtrm*, we generated *mtrm^126^ +/+ endos^00003^* females. Indeed, the fertility of these double heterozygous females was significantly reduced relative to the fertility of *mtrm^126^/+* or *endos^00003^/+* single heterozygous females (see Figure 4A).

### *endos* and *mtrm* do not dominantly interact to control meiosis

**Figure 3:**
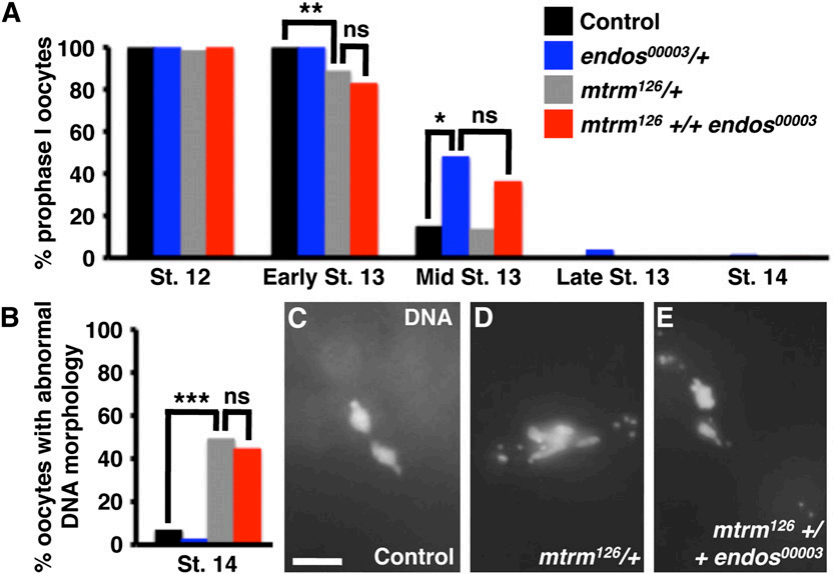
*endos* and *mtrm* do not show genetic interactions during oocyte meiosis. (A) Quantification of percentage of oocytes remaining in prophase I at stages 12, early 13, mid 13, and late 13 and 14 of oogenesis. Numbers of oocytes analyzed for *y w* control, *endos^00003^/+*, *mtrm^126^/+*, and *mtrm^126^ +/+* *endos^00003^* are as follows, respectively. Stage 12: 69, 56, 68, 41; early stage 13: 101, 163, 171, 109; mid stage 13: 27, 92, 65, 61; late stage 13: 128, 247, 189, 265; stage 14: 106, 239, 312, 773. (B) Quantification of percentage of stage 14 oocytes showing abnormal DNA morphology. Numbers of oocytes analyzed for *y w* control, *endos^00003^/+*, *mtrm^126^/+*, and *mtrm^126^ +/+* *endos^00003^* are 106, 239, 312, and 773, respectively. ^*^P = 0.002. ^**^P = 0.0005. ^***^P = 1 × 10^−14^. ns, no significant difference. (C–E) Examples of stage 14 oocyte DNA morphology for control (C, normal), *mtrm^126^* (D, abnormal) and *mtrm^126^ +/+ endos^00003^* (E, abnormal). Scale bar, 5 μm.

The reduced fertility of *mtrm^126^ +/+ endos^00003^* females could result from defects in oocyte meiosis, early embryonic mitoses, or a combination of both. *endos^00003^* homozygous females have a prolonged prophase I arrest, and Endos positively regulates Polo protein expression levels (Von Stetina *et al.* 2008). *mtrm^126^/+* heterozygous females have premature meiotic maturation, and Mtrm binds to Polo and inhibits its activity (Xiang *et al.* 2007). Analyzing DNA morphology as previously described (Von Stetina *et al.* 2008; see Figure 1), we observed that a small but significant percentage of oocytes from *mtrm^126^/+* females are released from the prophase I arrest slightly prematurely (see “Early St. 13” in Figure 3A); this result is consistent with but not as pronounced as previously reported (Xiang *et al.* 2007). Conversely, a fraction of *endos^00003^/+* heterozygous oocytes shows prolonged prophase I arrest (see “Mid St. 13” in Figure 3A); this phenotype is in agreement with the more severe prolongation of prophase I in *endos^00003^* homozygotes (Von Stetina *et al.* 2008). *mtrm^126^ +/+ endos^00003^* double heterozygotes show an intermediate phenotype, with some oocytes leaving prophase I slightly prematurely and others remaining in prophase I longer (Figure 3A).

*endos^00003^* homozygous females do not progress to metaphase I and show a dispersed DNA morphology in stage 14 oocytes (Von Stetina *et al.* 2008), and *mtrm^126^/+* heterozygous stage 14 oocytes have mispositioned chromosomes, resulting in a high frequency of nondisjunction (Xiang *et al.* 2007). We therefore also examined genetic interactions between *endos* and *mtrm* in stage 14 oocytes. The vast majority of control and *endos^00003^*/+ heterozygous oocytes displayed typical metaphase I morphology (Figure 3, B and C; also see Figure 1B′, B′′). *mtrm^126^/+* heterozygotes displayed a high frequency of oocytes containing misarranged chromosome masses (Figure 3, B and D), consistent with reported non-disjunction defects (Xiang *et al.* 2007). Removal of one copy of *endos* did not suppress or enhance the abnormal stage 14 DNA morphology of *mtrm^126^/+* heterozygous oocytes (Figure 3, B and E). Taken together, these results suggest that *endos^00003^* and *mtrm^126^* do not have significant genetic interactions during meiotic maturation.

### Maternal *endos* and *mtrm* dominantly interact to control early embryonic mitoses

As described previously, the fertility of *mtrm^126^ +/+ endos^00003^* double-heterozygous females is markedly reduced relative to that of single heterozygotes. In accordance, embryos produced by *mtrm^126^ +/+ endos^00003^* females have a very reduced hatch rate (1.3%) relative to those produced by *y w* control (90%), *endos^00003^* heterozygous (94%), or *mtrm^126^* heterozygous (74%) females (Figure 4A). Because meiosis does not appear to be significantly disrupted in double heterozygotes (Figure 3), we instead examined the early mitoses of embryos laid by these females (Figure 4, B–D). Most of the embryos laid by *y w* control (91%) or *endos^00003^* heterozygous (95%) females had normal DNA and spindle morphology (Figure 4, B and C). A sizeable fraction of embryos from *mtrm^126^* heterozygous females had DNA morphology defects and abnormal spindles during early mitoses (61% normal; Figure 4B), consistent with the slightly reduced hatch rate of these embryos (Figure 4A). Early mitosis defects, however, were significantly more severe in embryos derived from *mtrm^126^ +/+ endos^00003^* double-heterozygous females (only 19% normal; Figure 4, B and D−G). These results indicate that *mtrm* and *endos* genetically interact in the maternal control of early embryonic mitoses.

*endos* and *mtrm* have distinct effects on Polo. *endos^00003^* homozygous females have reduced levels of Polo expression in stage 14 oocytes (Von Stetina *et al.* 2008), and Mtrm is thought to bind to Polo stoichiometrically and inhibit its activity (Xiang *et al.* 2007). The effect of *mtrm* on *polo* function is dosage sensitive because *mtrm^126^*/+ heterozygous females have increased meiotic nondisjunction, and this phenotype is rescued by removal of one copy of *polo* and partially phenocopied by an extra copy of *polo* (Xiang *et al.* 2007). Accordingly, halving the dosage of *polo* also suppresses the decreased hatch rates of embryos derived from *mtrm^126^*/+ heterozygous females (Figure 4). We therefore reasoned that the strong genetic interaction between *endos* and *mtrm* during early embryonic mitoses might result from alterations in *polo* function. Indeed, removal of one copy of *polo* (*polo^16-1^*/+) in *mtrm^126^ +/+ endos^00003^* double-heterozygous females completely restored normal embryonic hatch rates and early mitoses (Figure 4, A, B, and H), whereas introducing one extra copy of *polo* (*GFP::polo; +/+*) resulted in very low hatch rates of embryos from both *mtrm^126^/+* and *mtrm^126^ +/+ endos^00003^* females (Figure 4, A and B). These results provide strong genetic evidence to support the model that early embryos produced by *mtrm^126^ +/+ endos^00003^* females have abnormally high Polo activity, leading to abnormal mitoses.

### Endos regulates the levels of Mtrm maternally loaded into the early embryo

**Figure 5:**
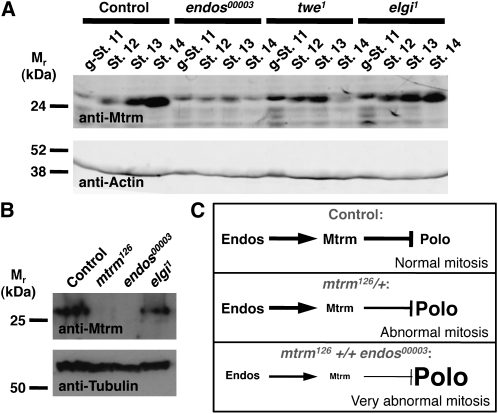
*endos* is required for maternal expression of Mtrm. (A) Western blot showing Mtrm protein expression at different stages of oogenesis in *y w* control, *endos^00003^*, *twe^1^*, or *elgi^1^* homozygous females. g-St. 11, germarium through stage 11; St. 12, stage 12; St. 13, stage 13; St. 14, stage 14. Actin was used as a loading control. One hundred egg chambers (or g-St.11 sets) were loaded per lane. (B) Mtrm Western blot of *y w* control, *mtrm^126^*, *endos^00003^*, and *elgi^1^* stage 14 oocytes. Tubulin was used as a loading control. We were unable to examine Mtrm levels in *elgi^1^ endos^00003^* double homozygous females due to the poor health of this genotype. (C) Model for genetic interaction between *endos* and *mtrm*. In wild-type females (Control), Endos promotes expression of Mtrm, a known negative regulator of Polo, during oogenesis resulting in normal levels of Polo function in the early embryo. In *mtrm^126^/+* heterozygous females, reduced levels of maternal Mtrm loaded into the embryo lead to increased Polo activity and abnormal embryonic mitoses. Removal of one copy of *endos* in *mtrm^126^ +/+ endos^00003^* double heterozygous females results in early embryos with further reduced levels of Mtrm, leading to even higher levels of Polo activity and more severe defects during early embryonic mitoses.

Our observations that maternal *mtrm* interacts with *endos* to control early embryonic mitoses were initially surprising because Mtrm expression had been reported to be greatly reduced by stage 13 of oogenesis, based on immunofluorescence assays (Xiang *et al.* 2007). Because antibody penetration often presents a challenge for immunofluorescence detection in later stages of oogenesis, we examined Mtrm expression by Western blotting analyses of staged egg chambers. In *y w* control females, Mtrm is very highly expressed in stages 13 and 14 of oogenesis (Figure 5A). The band recognized by the antibody indeed corresponds to Mtrm, as it is not present in *mtrm^126^* homozygous stage 14 oocytes (Figure 5B). These results indicate that Mtrm protein is maternally loaded into early embryos.

Removal of one copy of *endos* strongly enhances the early mitosis defects of embryos produced by *mtrm^126^/+* heterozygotes (see Figure 4), and Endos regulates the levels of several cell-cycle regulators in mature oocytes, including Polo, Twine (Twe), and Cyclin A (Von Stetina *et al.* 2008; J. R. Von Stetina and D. Drummond-Barbosa, unpublished data). We therefore tested whether *endos* might control the expression of Mtrm during oogenesis. In *endos^00003^* homozygous females, Mtrm levels in earlier stages of egg chamber development are comparable with those of controls. In contrast, *endos^00003^* homozygous stage 13 and 14 oocytes show markedly reduced levels of Mtrm (Figure 5, A and B).

Endos and Elgi, a predicted E3 ubiquitin ligase, physically interact *in vitro*, and antagonize each other *in vivo* (Von Stetina *et al.* 2008). Although *elgi* is not involved in the degradation of Polo or Twe (Von Stetina *et al.* 2008), we tested whether Elgi might control Mtrm levels. *elgi^1^* homozygous females, however, did not have increased levels of Mtrm in stages 13 and 14 (Figure 5, A and B). These results suggest that Elgi is not a major regulator of Mtrm levels in mature oocytes and that *endos* likely regulates Mtrm levels via a separate pathway. Indeed, we observed a reduction in Mtrm levels in oocytes of females mutant for *twe* (*twe^1^*; Figure 5A), which encodes a positive regulator of Cdk1, suggesting that the effects of *endos* on Mtrm expression might at least in part be a consequence of reduced phosphorylation of Cdk1 substrates. Based on our data, we speculate that *mtrm^126^ +/+ endos^00003^* double heterozygous females have sufficient Polo expression to complete meiosis. However, the lower *mtrm* gene dosage combined with the further reduction in Mtrm expression caused by *endos* heterozygosity substantially reduces the levels of this dose-sensitive stoichiometric regulator of Polo, leading to abnormally high Polo activity levels and severe early mitoses defects (Figure 5C).

## Discussion

We had previously identified *endos* as a key regulator of meiotic maturation and early embryonic mitoses (Von Stetina *et al.* 2008), and our genetic screen in this study identified several genomic intervals that dominantly interact with *endos^00003^/+* to produce strong sterility or viability defects. These results suggest that *endos* plays multiple roles during the cell cycle throughout development and that its effects are complex and dosage dependent. Evidence from its vertebrate homolog (Gharbi-Ayachi *et al.* 2010; Mochida *et al.* 2010) suggests that Endos may act as a small regulatory protein that represses PP2A to promote entry into mitosis and meiotic maturation, and a recent study provides genetic support for this model in *Drosophila* (Rangone *et al.* 2011). It is conceivable that Endos binds additional cell cycle proteins to modulate their activity in a similar fashion.

We uncovered *mtrm*, which encodes a stoichiometric inhibitor of Polo kinase, as a strong dominant enhancer of *endos*. Double heterozygosity of *endos* and *mtrm* induced severe sterility mostly as the result of defects in early embryonic mitoses. By reducing the gene dosage of *polo* we completely rescued the fertility defects of *mtrm^126^ +/+ endos^00003^* females, indicating that the primary cause of this sterility phenotype is excessive Polo activity. Although we previously showed that Endos positively controls Polo protein levels during meiosis (Von Stetina *et al.* 2008), these data suggest that later, during early embryonic mitoses, maternal *endos* antagonizes Polo activity through its effects on Mtrm expression during oocyte development (Figure 5C). We thus propose that Endos is required during late stages of oogenesis to promote high levels of Mtrm protein to be loaded into early embryos for the proper stoichiometric balance between Mtrm and Polo, which is essential for normal mitoses in early embryogenesis (Figure 5C).

The early syncytial embryo is particularly sensitive to the balance between specific cell-cycle regulators. For example, a balance between Gwl kinase and Polo activities has been proposed to be crucial for early embryonic mitoses (Archambault *et al.* 2007). Embryos derived from females heterozygous for both *polo* and *Scant*, a putative hyperactive allele of *gwl*, are not viable because of a significant loss or detachment of centrosomes, and this phenotype is rescued by increased maternal *polo* dosage. These studies led to the conclusion that excessive Gwl activity relative to Polo results in defects in the early syncytial embryo. Interestingly, the authors proposed that phosphorylation of an intermediate substrate by Gwl mediates its inhibitory effect since Gwl does not phosphorylate Polo (Archambault *et al.* 2007). Given that vertebrate Endos is phosphorylated and activated by Gwl (Gharbi-Ayachi *et al.* 2010; Mochida *et al.* 2010), and this also appears to be the case in *Drosophila* (Rangone *et al.* 2011), it is tempting to speculate that Gwl acts upstream of Endos to control Mtrm expression during late oogenesis and thereby antagonizes Polo in the early embryo.

Endos controls the expression of multiple key regulators of the cell cycle, including Polo, Twe/Cdc25, and Cyclin A (Von Stetina *et al.* 2008; J. R. Von Stetina and D. Drummond-Barbosa, unpublished data), and in this study we found that Mtrm expression also requires *endos* function. Although it is formally possible that Endos might control the expression of other proteins via the predicted E3 ubiquitin ligase Elgi, Elgi does not appear to mediate the degradation of Polo, Twe or Mtrm in *endos* mutant females. Instead, it is conceivable that *endos* might regulate the activity of the anaphase-promoting complex/cyclosome (APC/C), which controls the degradation of multiple cell-cycle regulators (Peters 2006). Future studies should address functional interactions between the anaphase-promoting complex/cyclosome and *endos*.

Our data uncovering a zygotic role for *endos* suggests that it may not only function during meiosis or specialized S-M early embryonic mitoses but also act as a general regulator of the cell-cycle machinery. This idea is consistent with a recent study showing that *endos* is zygotically required during *Drosophila* development for neuroblast proliferation (Rangone *et al.* 2011), and with the recently identified roles for *endos* in chromosome alignment and spindle assembly (Goshima *et al.* 2007), the G2-M DNA damage checkpoint (Kondo and Perrimon 2011), and with the PP2A inhibitory function of vertebrate Endos (Gharbi-Ayachi *et al.* 2010; Mochida *et al.* 2010). The molecular identification of the specific genes interacting with *endos* in both sterile and lethal combinations will help us better understand how Endos controls the cell-cycle machinery and how Endos is regulated during the cell cycle. Based on the evolutionary conservation of Endos and its cell-cycle functions, this knowledge will likely be of broad relevance.

## Supplementary Material

Supporting Information
