# Supplementary material for: A Genetic Screen for Dominant Enhancers of the Cell-Cycle Regulator α-Endosulfine Identifies Matrimony as a Strong Functional Interactor in Drosophila
Source: G3 (Bethesda). 2011 Dec 1;1(7):607–13. doi: 10.1534/g3.111.001438 (PMC3276179; doi:10.1534/g3.111.001438)
Supplement: Supporting Information [file supp_1_7_607__index.html]

Supporting Information 

# A Genetic Screen for Dominant Enhancers of the Cell-Cycle Regulator α-Endosulfine Identifies Matrimony as a Strong Functional Interactor in *Drosophila*

## Supporting Information for Von Stetina *et al.*, 2011

**Files in this Data Supplement:**

- Supporting Information - Figure S1, File S1, and Tables S1-S3 (PDF, 472 KB)
- Figure S1 - F1 screen for deficiencies that dominantly enhance *endos00003*/+ leading to female sterility or lethality (PDF, 140 KB)
- File S1 - List of deficiencies tested in F1 screen for *endos00003* dominant enhancers (PDF, 52 KB)
- Table S1 - Additional deficiencies tested for *endos00003*-interacting genomic regions with reduced fertility phenotype(PDF, 140 KB)
- Table S2 - Deficiencies that cause zygotic lethality of *endos00003*/+ heterozygotes (PDF, 48 KB)
- Table S3 - Additional deficiencies tested for *endos00003*-interacting genomic regions with lethality phenotype (PDF, 108 KB)
