# Supplementary material for: A Genetic Screen for Dominant Enhancers of the Cell-Cycle Regulator α-Endosulfine Identifies Matrimony as a Strong Functional Interactor in Drosophila
Source: G3 (Bethesda). 2011 Dec 1;1(7):607–13. doi: 10.1534/g3.111.001438 (PMC3276179; doi:10.1534/g3.111.001438)
Supplement: Supporting Information [file supp_1.7.607_001438SI.pdf]

File S1

List of deficiencies tested in F1 screen for *endos*^{00003} dominant enhancers

Chromosome 1: Df(1)N-8, Df(1)64c18, Df(1)A113, Df(1)JC70, Df(1)ct-J4, Df(1)RA2, Df(1)KA14, Df(1)C52, Df(1)HA85, Df(1)N105, Df(1)JA27, Df(1)DCB1-35b, Df(1)RK2, Df(1)BA1, Df(1)sc-J4, Df(1)XR38, Df(1)Sxl-bt, Df(1)B25, Df(1)BK10, Df(1)dx81, Df(1)RR79, In(1)AC2^{L}*AB*^{R}*, Df(1)Exel6253, Df(1)Exel6291, Df(1)ED6878, Df(1)ED7364.*

Chromosomal arm 2L: Df(2L)C144, Df(2L)TE29Aa-11, Df(2L)TW137, Df(2L)E110, Df(2L)cl-h3, Df(2L)r10, Df(2L)JS17, Df(2L)spd^{j2}*, Df(2L)cact-255rv64, Df(2L)N22-14, Df(2L)Prl, Df(2L)ast2, Df(2L)dp-79b, Df(2L)b87e25, Df(2L)J2, Df(2L)TE35BC-24, Df(2L)net-PMF, Df(2L)XE-3801*, Df(2L)C', *Df(2L)ed1, Df(2L)Dwee1-W05, Df(2L)BSC4, Df(2L)BSC5, Df(2L)BSC6, Df(2L)BSC7, Df(2L)BSC17, Df(2L)BSC16, Df(2L)dpp*^{d14}*, Df(2L)BSC28, Df(2L)BSC30, Df(2L)BSC31, Df(2L)BSC36, Df(2L)BSC37, Df(2L)BSC41, Df(2L)Exel6011, Df(2L)Exel6049, Df(2L)BSC50, Df(2L)BSC106, Df(2L)BSC109, Df(2L)BSC110, Df(2L)BSC111, Df(2L)ED250, Df(2L)ED611, Df(2L)BSC142, Df(2L)BSC143, Df(2L)BSC145, Df(2L)BSC147, Df(2L)BSC151.*

Chromosomal arm 2R: Df(2R)H3E1, Df(2R)X58-12, Df(2R)CX1, Df(2R)M41A4, In(2R)bw^{VDe2L} *Cy*^{R}*, Df(2R)vg-C, Df(2R)P34, Df(2R)nap9, Df(2R)en30, Df(2R)PC4, Df(2R)or-BR6, Df(2R)X1, Df(2R)B5, Df(2R)ST1, Df(2R)M60E, Df(2R)Px2, Df(2R)AA21, Df(2R)Jp1, Df(2R)Jp8, Df(2R)Np5, Df(2R)59AD, Df(2R)Kr10, Df(2R)w45-30n, Df(2R)Egfr5, Df(2R)robl-c, Df(2R)BSC3, Df(2R)BSC11, Df(2R)BSC18, Df(2R)BSC22, Df(2R)14H10Y-53, Df(2R)14H10W-35, Df(2R)BSC26, Df(2R)BSC29, Df(2R)BSC39, Df(2R)BSC40, Df(2R)vir130, Df(2R)BSC45, Df(2R)Exel7130, Df(2R)Exel7131, Df(2R)ED4065, Df(2R)BSC132, Df(2R)BSC134, Df(2R)BSC161, Df(2R)BSC155, Df(2R)BSC550.*

Chromosomal arm 3L: Df(3L)GN34, Df(3L)pbl-X1, Df(3L)66C-G28, Df(3L)rdgC-co2, Df(3L)R-G7, Df(3L)emc-E12, Df(3L)vin5, Df(3L)vin7, Df(3L)st-f13, Df(3L)h-i22, Df(3L)ZN47, Df(3L)fz-GF3b, Df(3L)ri-79c, Df(3L)kto2, Df(3L)brm11, Df(3L)HR119, In(3LR)C190^{L}*Ubx*^{42TR}*, Df(3L)XDI98, Df(3L)Pc-2q, Df(3L)Scf-R6, Df(3L)Ten-m-AL29, Df(3L)XS533, Df(3L)eyg*^{C1}*, Df(3L)ZP1, Df(3L)ri-XT1, Df(3L)HD1, Df(3L)BSC10, Df(3L)BSC12, Df(3L)BSC14, Df(3L)XG5, Df(3L)BSC21, Df(3L)fz2, Df(3L)BSC33, Df(3L)BSC35, Df(3L)Exel6087, Df(3L)ED4782, Df(3L)ED4978, Df(3L)BSC181, Df(3L)BSC223, Df(3L)BSC249, Df(3L)BSC283.*

Chromosomal arm 3R: Df(3R)ea, Df(3R)3450, Df(3R)Dr-rv1, Df(3R)D605, Df(3R)P115, Df(3R)ME15, Tp(3;Y)ry506-85C, Df(3R)Scr, Df(3R)Tl-P, Df(3R)by10, Df(3R)p712, Df(3R)Tpl10, Df(3R)crb87-5, Df(3R)WIN11, Df(3R)mbc-R1, Df(3R)23D1, Df(3R)T-32, Df(3R)Cha7, Df(3R)Dl-BX12, Df(3R)M-Kx1, Df(3R)e-R1, Df(3R)B81, Df(3R)DG2, Df(3R)crb-F89-4, Df(3R)mbc-30, Df(3R)H-B79, Df(3R)Espl3, Df(3R)e1025-14, Df(3R)BSC24, Df(3R)BSC38, Df(3R)BSC42, Df(3R)BSC43, Df(3R)BSC47, Df(3R)Exel6144, Df(3R)Exel6195, Df(3R)Exel6196, Df(3R)Exel6197, Df(3R)Exel6202, Df(3R)Exel6203, Df(3R)Exel9012, Df(3R)Exel9014, Df(3R)ED5177, Df(3R)BSC55, Df(3R)BSC56, Df(3R)BSC137, Df(3R)BSC140, Df(3R)IR16.

Chromosome 4: Df(4)O2.

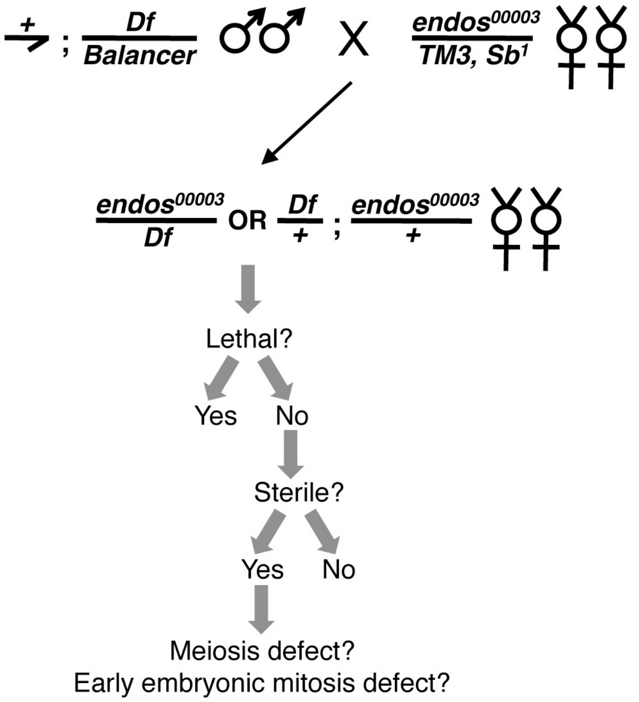

Figure S1 F1 screen for deficiencies that dominantly enhance *endos*^{00003}*/+* leading to female sterility or lethality. Males carrying a balanced deficiency (*Df*) were mated to *endos*^{00003} heterozygous (*endos*^{00003}*/TM3, Sb*^{1}) virgin females. The genetic interaction was considered lethal if progeny consisted exclusively of flies carrying balancer chromosomes. If adult balancer-free progeny were present, *endos*^{00003}*/Df* or *Df/+; endos*^{00003}*/+* females were tested for their fertility by mating to wild-type males. If these females produced reduced or absent progeny, further analyses were conducted to test for meiotic maturation or early embryonic mitosis defects.

Table S1 Additional deficiencies tested for *endos*^{00003}-interacting genomic regions with reduced fertility phenotype

Deficiency[a]	Deleted segment[b]	Likely location of interacting gene
Df(1)JC70[c]	4C11—5A4	4F1—4F4
Df(1)BSC533	4F4—4F10	
Df(1)Exel6290	4F7—4F10	
Df(1)ovo41	4C6—4F1	
Df(2)J2[c]	31B1—32A2	31D9—31E1
Df(2L)Exel9032	31A3—31B1	
Df(2L)BSC144	31B1—31E5	
Df(2L)ED8142	31E1—32A4	
Df(2L)BSC32	32A1—32D1	
Df(2L)BSC342	31D9—31E5	
Df(2L)J3	31D1—31F5	
Df(2R)X1[c]	46C2—47A1	Unknown[d]
Df(2R)BSC133	46B4—46C1	
Df(2R)X3	46C1—46E2	
Df(2R)12	46F—47A10	
Df(2R)BSC298	46B2—46C7	
Df(2R)CX1[c]	49C1—50D5	Unknown[d]
Df(2R)ED2308	49C3—49E7	
Df(2R)Exel7124	49F10—50A1	
Df(2R)BSC273	49F4—50A13	
Df(2R)BSC274	50A7—50B4	
Df(2R)BSC307	50B6—50C18	
Df(2R)BSC361	50C3—50F1	
Df(2R)Exel6062	49E6—49F1	
Df(2R)Exel8057	49E6—49F10	
Df(2R)BSC485	49B10—49E6	
Df(2R)BSC11[c]	50E6—51E4	51A2—51A4
Df(2R)BSC357	50F6—51C1	
Df(2R)L48	50F6—51B3	
Df(2R)Exel6284	51B1—51C2	
Df(2R)KnSA3	51B5—51D1	
Df(2R)Jp1	51C3—52F9	
Df(2R)Exel8059	51A4—51B1	
Df(2R)BSC668	51A2—51C1	
Df(3R)WIN11[c]	83E1—84A5	84A5

Df(3R)Scr[c]	84A1—84B2	
Df(3R)Dfd13	83E3—84B1	
Df(3R)BSC467	83F1—84B2	
Df(3R)BD5	84A1—84B2	
Df(3R)BSC422	84A5—84B2	
Df(3R)LIN	84A5—84B1	
Df(3R)roe	84A6—84D9	
Df(3R)pb-X2	84A4—84B2	
Df(3R)by10[c]	85D8—85E13	85D10—85D24
Df(3R)by416	85D10—85E2	
Df(3R)BSC526	85E8—85F14	
Df(3R)Exel6264	85D24—85E5	
Df(3R)BSC528	85E1	
Df(3R)BSC468	85E1—85E4	
Df(3R)BSC43[c]	92F7—93B6	92F7—92F13
Df(3R)BSC518	92E8—92F13	
Df(3R)BSC680	93A2—93B8	
Df(3R)e-N19	93B2—94A8	
Df(3R)Exel6185	92E2—92F1	
Df(3R)23D1[c]	94A3—94D4	94C4
Df(3R)ED6093	94A2—94C4	
Df(3R)ED6096	94B5—94E7	
Df(3R)BSC618	94C4—94E3	
Df(3R)BSC55	94D2—94E6	
Df(3R)Exel6193	94D3—94E4	
Df(3R)ED6103	94D3—94E9	

[a] Deficiencies in bold-type result in reduced fertility when *in trans* to *endos*^{00003}; other deficiencies show no genetic interaction with *endos*^{00003}.
[b] Deleted genomic region represented according to polytene chromosome divisions (http://flybase.org).
[c] Deficiencies used in the original deficiency screen for *endos*^{00003} enhancers.
[d] Results with multiple deficiencies in the interacting region are inconsistent.

Table S2 Deficiencies that cause zygotic lethality of *endos*^{00003}/+ heterozygotes

Deficiency	Deleted segment[a]
Df(2R)Kr10	60E10—60F5
Df(3L)h-i22	66D10—66E2
Df(3L)st-f13	72C1—73A4
Tp(3;Y)ry506-85C	87D1—88E6
Df(3R)e-R1	93B6—93D4

[a] Deleted genomic region represented according to polytene chromosome divisions (http://flybase.org).

Table S3 Additional deficiencies tested for *endos*^{00003}-interacting genomic regions with lethality phenotype

Deficiency[a]	Deleted segment[b]	Likely location of interacting gene
Df(2R)Kr10[c]	60E10—60F5	60F1—60F5
Df(2R)M60E	60E6—60E11	
Df(2R)gsb	60E9—60F1	
Df(2R)ED50004	60F5	
Df(3L)h-i22[c]	66D10—66E2	66D12
Df(3L)ED4414	66D12—66E6	
Df(3L)ED4421	66D12—67B3	
Df(3L)ED4416	66E1—67B1	
Df(3L)Scf-R11	66E3—66F2	
Df(3L)st-f13[c]	72C1—73A4	72D1—72D4
Df(3L)st-g24	72D1—73A10	
Df(3L)4606	72D4—73C4	
Df(3L)st-b11	72D10—73D2	
Df(3L)ED223	73A1—73D5	
Df(3L)81k19	73A3—74F4	
Tp(3;Y)ry506-85C[c]	87D1—88E6	88D1
Df(3R)ry615	87B10—87E8	
Df(3R)ry85	87B15—88A1	
Df(3R)ED5612	87C7—87F6	
Df(3R)ED5623	87E3—88A4	
Df(3R)MRS	87E8—93C	
Df(3R)ED5642	87F10—88C2	
Df(3R)ED5644	88A4—88C9	
Df(3R)red1	88A2—88D1	
Df(3R)ea	88E7—89A1	
Df(3R)ED5664	88D1—88E3	
Df(3R)Exel6275	88D1—88D7	
Df(3R)BSC635	88D2—88E3	
Df(3R)Exel6172	88D5—88D7	
Df(3R)Exel6173	88D7—88E1	
Df(3R)ED10566	88D6—88E1	
Df(3R)BSC750	88E2—88E5	
Df(3R)e-R1[c]	93B6—93D4	93C1—93D4
Df(3R)ED10838	93C1—93D4	
Df(3R)ED6058	93D4—93F6	

[a] Deficiencies in bold-type result in lethality when *in trans* to *endos*^{00003}; other deficiencies show no genetic interaction with *endos*^{00003}.
[b] Deleted genomic region represented according to polytene chromosome divisions (http://flybase.org).
[c] Deficiencies used in the original deficiency screen for *endos*^{00003} enhancers.
