# Supplementary material for: A Genetic Screen for Dominant Enhancers of the Cell-Cycle Regulator α-Endosulfine Identifies Matrimony as a Strong Functional Interactor in Drosophila
Source: G3 (Bethesda). 2011 Dec 1;1(7):607–13. doi: 10.1534/g3.111.001438 (PMC3276179; doi:10.1534/g3.111.001438)
Supplement: Supporting Information [file supp_1.7.607_FigureS1.pdf]

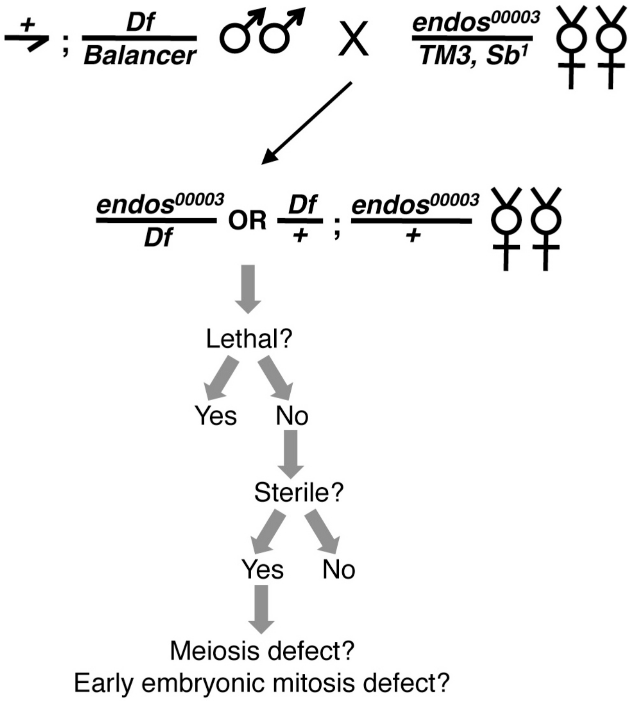

Figure S1 F1 screen for deficiencies that dominantly enhance $endos^{00003}$/+ leading to female sterility or lethality. Males carrying a balanced deficiency (*Df*) were mated to $endos^{00003}$ heterozygous ($endos^{00003}$/*TM3, Sb*^{1}) virgin females. The genetic interaction was considered lethal if progeny consisted exclusively of flies carrying balancer chromosomes. If adult balancer-free progeny were present, $endos^{00003}$/*Df* or *Df*/+; $endos^{00003}$/+ females were tested for their fertility by mating to wild-type males. If these females produced reduced or absent progeny, further analyses were conducted to test for meiotic maturation or early embryonic mitosis defects.
