# Supplementary material for: A Genetic Screen for Dominant Enhancers of the Cell-Cycle Regulator α-Endosulfine Identifies Matrimony as a Strong Functional Interactor in Drosophila
Source: G3 (Bethesda). 2011 Dec 1;1(7):607–13. doi: 10.1534/g3.111.001438 (PMC3276179; doi:10.1534/g3.111.001438)
Supplement: Supporting Information [file supp_1.7.607_FileS1.pdf]

File S1

List of deficiencies tested in F1 screen for *endos*^{00003} dominant enhancers

Chromosome 1: Df(1)N-8, *Df(1)64c18*, *Df(1)A113*, *Df(1)JC70*, *Df(1)ct-J4*, *Df(1)RA2*, *Df(1)KA14*, *Df(1)C52*, *Df(1)HA85*, *Df(1)N105*, *Df(1)JA27*, *Df(1)DCB1-35b*, *Df(1)RK2*, *Df(1)BA1*, *Df(1)sc-J4*, *Df(1)XR38*, *Df(1)Sxl-bt*, *Df(1)B25*, *Df(1)BK10*, *Df(1)dx81*, *Df(1)RR79*, *In(1)AC2*^{L}*AB*^{R}, *Df(1)Exel6253*, *Df(1)Exel6291*, *Df(1)ED6878*, *Df(1)ED7364*.

Chromosomal arm 2L: Df(2L)C144, *Df(2L)TE29Aa-11*, *Df(2L)TW137*, *Df(2L)E110*, *Df(2L)cl-h3*, *Df(2L)r10*, *Df(2L)JS17*, *Df(2L)spd*^{j2}, *Df(2L)cact-255rv64*, *Df(2L)N22-14*, *Df(2L)Prl*, *Df(2L)ast2*, *Df(2L)dp-79b*, *Df(2L)b87e25*, *Df(2L)J2*, *Df(2L)TE35BC-24*, *Df(2L)net-PMF*, *Df(2L)XE-3801*, Df(2L)C’, *Df(2L)ed1*, *Df(2L)Dwee1-W05*, *Df(2L)BSC4*, *Df(2L)BSC5*, *Df(2L)BSC6*, *Df(2L)BSC7*, *Df(2L)BSC17*, *Df(2L)BSC16*, *Df(2L)dpp*^{d14}, *Df(2L)BSC28*, *Df(2L)BSC30*, *Df(2L)BSC31*, *Df(2L)BSC36*, *Df(2L)BSC37*, *Df(2L)BSC41*, *Df(2L)Exel6011*, *Df(2L)Exel6049*, *Df(2L)BSC50*, *Df(2L)BSC106*, *Df(2L)BSC109*, *Df(2L)BSC110*, *Df(2L)BSC111*, *Df(2L)ED250*, *Df(2L)ED611*, *Df(2L)BSC142*, *Df(2L)BSC143*, *Df(2L)BSC145*, *Df(2L)BSC147*, *Df(2L)BSC151*.

Chromosomal arm 2R: Df(2R)H3E1, *Df(2R)X58-12*, *Df(2R)CX1*, *Df(2R)M41A4*, *In(2R)bw*^{VDe2L} *Cy*^{R}, *Df(2R)vg-C*, *Df(2R)P34*, *Df(2R)nap9*, *Df(2R)en30*, *Df(2R)PC4*, *Df(2R)or-BR6*, *Df(2R)X1*, *Df(2R)B5*, *Df(2R)ST1*, *Df(2R)M60E*, *Df(2R)Px2*, *Df(2R)AA21*, *Df(2R)Jp1*, *Df(2R)Jp8*, *Df(2R)Np5*, *Df(2R)59AD*, *Df(2R)Kr10*, *Df(2R)w45-30n*, *Df(2R)Egfr5*, *Df(2R)robl-c*, *Df(2R)BSC3*, *Df(2R)BSC11*, *Df(2R)BSC18*, *Df(2R)BSC22*, *Df(2R)14H10Y-53*, *Df(2R)14H10W-35*, *Df(2R)BSC26*, *Df(2R)BSC29*, *Df(2R)BSC39*, *Df(2R)BSC40*, *Df(2R)vir130*, *Df(2R)BSC45*, *Df(2R)Exel7130*, *Df(2R)Exel7131*, *Df(2R)ED4065*, *Df(2R)BSC132*, *Df(2R)BSC134*, *Df(2R)BSC161*, *Df(2R)BSC155*, *Df(2R)BSC550*.

Chromosomal arm 3L: Df(3L)GN34, *Df(3L)pbl-X1*, *Df(3L)66C-G28*, *Df(3L)rdgC-co2*, *Df(3L)R-G7*, *Df(3L)emc-E12*, *Df(3L)vin5*, *Df(3L)vin7*, *Df(3L)st-f13*, *Df(3L)h-i22*, *Df(3L)ZN47*, *Df(3L)fz-GF3b*, *Df(3L)ri-79c*, *Df(3L)kto2*, *Df(3L)brm11*, *Df(3L)HR119*, *In(3LR)C190*^{L}*Ubx*^{42TR}, *Df(3L)XDI98*, *Df(3L)Pc-2q*, *Df(3L)Scf-R6*, *Df(3L)Ten-m-AL29*, *Df(3L)XS533*, *Df(3L)eyg*^{C1}, *Df(3L)ZP1*, *Df(3L)ri-XT1*, *Df(3L)HD1*, *Df(3L)BSC10*, *Df(3L)BSC12*, *Df(3L)BSC14*, *Df(3L)XG5*, *Df(3L)BSC21*, *Df(3L)fz2*, *Df(3L)BSC33*, *Df(3L)BSC35*, *Df(3L)Exel6087*, *Df(3L)ED4782*, *Df(3L)ED4978*, *Df(3L)BSC181*, *Df(3L)BSC223*, *Df(3L)BSC249*, *Df(3L)BSC283*.

Chromosomal arm 3R: Df(3R)ea, *Df(3R)3450*, *Df(3R)Dr-rv1*, *Df(3R)D605*, *Df(3R)P115*, *Df(3R)ME15*, *Tp(3;Y)ry506-85C*, *Df(3R)Scr*, *Df(3R)Tl-P*, *Df(3R)by10*, *Df(3R)p712*, *Df(3R)Tpl10*, *Df(3R)crb87-5*, *Df(3R)WIN11*, *Df(3R)mbc-R1*, *Df(3R)23D1*, *Df(3R)T-32*, *Df(3R)Cha7*, *Df(3R)Dl-BX12*, *Df(3R)M-Kx1*, *Df(3R)e-R1*, *Df(3R)B81*, *Df(3R)DG2*, *Df(3R)crb-F89-4*, *Df(3R)mbc-30*, *Df(3R)H-B79*, *Df(3R)Espl3*, *Df(3R)e1025-14*, *Df(3R)BSC24*, *Df(3R)BSC38*, *Df(3R)BSC42*, *Df(3R)BSC43*, *Df(3R)BSC47*, *Df(3R)Exel6144*, *Df(3R)Exel6195*, *Df(3R)Exel6196*, *Df(3R)Exel6197*, *Df(3R)Exel6202*, *Df(3R)Exel6203*, *Df(3R)Exel9012*, *Df(3R)Exel9014*, *Df(3R)ED5177*, *Df(3R)BSC55*, *Df(3R)BSC56*, *Df(3R)BSC137*, *Df(3R)BSC140*, *Df(3R)IR16*.

Chromosome 4: Df(4)O2.
