# Supplementary material for: A Genetic Screen for Dominant Enhancers of the Cell-Cycle Regulator α-Endosulfine Identifies Matrimony as a Strong Functional Interactor in Drosophila
Source: G3 (Bethesda). 2011 Dec 1;1(7):607–13. doi: 10.1534/g3.111.001438 (PMC3276179; doi:10.1534/g3.111.001438)
Supplement: Supporting Information [file supp_1.7.607_TableS1.pdf]

Table S1 Additional deficiencies tested for *endos*^{00003}-interacting genomic regions with reduced fertility phenotype

Deficiency[a]	Deleted segment[b]	Likely location of interacting gene
Df(1)JC70[c]	4C11—5A4	4F1—4F4
Df(1)BSC533	4F4—4F10	
Df(1)Exel6290	4F7—4F10	
Df(1)ovo41	4C6—4F1	
Df(2)J2[c]	31B1—32A2	31D9—31E1
Df(2L)Exel9032	31A3—31B1	
Df(2L)BSC144	31B1—31E5	
Df(2L)ED8142	31E1—32A4	
Df(2L)BSC32	32A1—32D1	
Df(2L)BSC342	31D9—31E5	
Df(2L)J3	31D1—31F5	
Df(2R)X1[c]	46C2—47A1	Unknown[d]
Df(2R)BSC133	46B4—46C1	
Df(2R)X3	46C1—46E2	
Df(2R)12	46F—47A10	
Df(2R)BSC298	46B2—46C7	
Df(2R)CX1[c]	49C1—50D5	Unknown[d]
Df(2R)ED2308	49C3—49E7	
Df(2R)Exel7124	49F10—50A1	
Df(2R)BSC273	49F4—50A13	
Df(2R)BSC274	50A7—50B4	
Df(2R)BSC307	50B6—50C18	
Df(2R)BSC361	50C3—50F1	
Df(2R)Exel6062	49E6—49F1	
Df(2R)Exel8057	49E6—49F10	
Df(2R)BSC485	49B10—49E6	
Df(2R)BSC11[c]	50E6—51E4	51A2—51A4
Df(2R)BSC357	50F6—51C1	
Df(2R)L48	50F6—51B3	
Df(2R)Exel6284	51B1—51C2	
Df(2R)KnSA3	51B5—51D1	
Df(2R)Jp1	51C3—52F9	
Df(2R)Exel8059	51A4—51B1	
Df(2R)BSC668	51A2—51C1	
Df(3R)WIN11[c]	83E1—84A5	84A5

Df(3R)Scr[c]	84A1—84B2	
Df(3R)Dfd13	83E3—84B1	
Df(3R)BSC467	83F1—84B2	
Df(3R)BD5	84A1—84B2	
Df(3R)BSC422	84A5—84B2	
Df(3R)LIN	84A5—84B1	
Df(3R)roe	84A6—84D9	
Df(3R)pb-X2	84A4—84B2	
Df(3R)by10[c]	85D8—85E13	85D10—85D24
Df(3R)by416	85D10—85E2	
Df(3R)BSC526	85E8—85F14	
Df(3R)Exel6264	85D24—85E5	
Df(3R)BSC528	85E1	
Df(3R)BSC468	85E1—85E4	
Df(3R)BSC43[c]	92F7—93B6	92F7—92F13
Df(3R)BSC518	92E8—92F13	
Df(3R)BSC680	93A2—93B8	
Df(3R)e-N19	93B2—94A8	
Df(3R)Exel6185	92E2—92F1	
Df(3R)23D1[c]	94A3—94D4	94C4
Df(3R)ED6093	94A2—94C4	
Df(3R)ED6096	94B5—94E7	
Df(3R)BSC618	94C4—94E3	
Df(3R)BSC55	94D2—94E6	
Df(3R)Exel6193	94D3—94E4	
Df(3R)ED6103	94D3—94E9	

[a] Deficiencies in bold-type result in reduced fertility when *in trans* to *endos*^{00003}; other deficiencies show no genetic interaction with *endos*^{00003}.
[b] Deleted genomic region represented according to polytene chromosome divisions (http://flybase.org).
[c] Deficiencies used in the original deficiency screen for *endos*^{00003} enhancers.
[d] Results with multiple deficiencies in the interacting region are inconsistent.
