# Supplementary material for: A Genetic Screen for Dominant Enhancers of the Cell-Cycle Regulator α-Endosulfine Identifies Matrimony as a Strong Functional Interactor in Drosophila
Source: G3 (Bethesda). 2011 Dec 1;1(7):607–13. doi: 10.1534/g3.111.001438 (PMC3276179; doi:10.1534/g3.111.001438)
Supplement: Supporting Information [file supp_1.7.607_TableS2.pdf]

Table S2 Deficiencies that cause zygotic lethality of *endos*^{00003}/+ heterozygotes

Deficiency	Deleted segment[a]
Df(2R)Kr10	60E10—60F5
Df(3L)h-i22	66D10—66E2
Df(3L)st-f13	72C1—73A4
Tp(3;Y)ry506-85C	87D1—88E6
Df(3R)e-R1	93B6—93D4

[a] Deleted genomic region represented according to polytene chromosome divisions (http://flybase.org).
