# Supplementary material for: A Genetic Screen for Dominant Enhancers of the Cell-Cycle Regulator α-Endosulfine Identifies Matrimony as a Strong Functional Interactor in Drosophila
Source: G3 (Bethesda). 2011 Dec 1;1(7):607–13. doi: 10.1534/g3.111.001438 (PMC3276179; doi:10.1534/g3.111.001438)
Supplement: Supporting Information [file supp_1.7.607_TableS3.pdf]

Table S3 Additional deficiencies tested for *endos*[00003]-interacting genomic regions with lethality phenotype

Deficiency[a]	Deleted segment[b]	Likely location of interacting gene
Df(2R)Kr10[c]	60E10—60F5	60F1—60F5
Df(2R)M60E	60E6—60E11	
Df(2R)gsb	60E9—60F1	
Df(2R)ED50004	60F5	
Df(3L)h-i22[c]	66D10—66E2	66D12
Df(3L)ED4414	66D12—66E6	
Df(3L)ED4421	66D12—67B3	
Df(3L)ED4416	66E1—67B1	
Df(3L)Scf-R11	66E3—66F2	
Df(3L)st-f13[c]	72C1—73A4	72D1—72D4
Df(3L)st-g24	72D1—73A10	
Df(3L)4606	72D4—73C4	
Df(3L)st-b11	72D10—73D2	
Df(3L)ED223	73A1—73D5	
Df(3L)81k19	73A3—74F4	
Tp(3;Y)ry506-85C[c]	87D1—88E6	88D1
Df(3R)ry615	87B10—87E8	
Df(3R)ry85	87B15—88A1	
Df(3R)ED5612	87C7—87F6	
Df(3R)ED5623	87E3—88A4	
Df(3R)MRS	87E8—93C	
Df(3R)ED5642	87F10—88C2	
Df(3R)ED5644	88A4—88C9	
Df(3R)red1	88A2—88D1	
Df(3R)ea	88E7—89A1	
Df(3R)ED5664	88D1—88E3	
Df(3R)Exel6275	88D1—88D7	
Df(3R)BSC635	88D2—88E3	
Df(3R)Exel6172	88D5—88D7	
Df(3R)Exel6173	88D7—88E1	
Df(3R)ED10566	88D6—88E1	
Df(3R)BSC750	88E2—88E5	
Df(3R)e-R1[c]	93B6—93D4	93C1—93D4
Df(3R)ED10838	93C1—93D4	
Df(3R)ED6058	93D4—93F6	

[a] Deficiencies in bold-type result in lethality when *in trans* to *endos*^{00003}; other deficiencies show no genetic interaction with *endos*^{00003}.
[b] Deleted genomic region represented according to polytene chromosome divisions (http://flybase.org).
[c] Deficiencies used in the original deficiency screen for *endos*^{00003} enhancers.
